# Smart governance and its role in achieving digital transformation requirements: A survey study in selected Iraqi universities

**DOI:** 10.12688/f1000research.174187.1

**Published:** 2026-04-16

**Authors:** Ahmed Majeed Ghareeb, Muhamed Khamis Sami Alisawi, Yousif Obed Hama Amin, Younis M. Kh. Al-Sabaawe, Ahmed Abbas Hammadi, Sara Hamid diab

**Affiliations:** 1 University of Fallujah, Al-Fallujah, Al Anbar Governorate, Iraq; 2 University of Human Development, Sulaymaniyah, Kurdistan Region, Iraq; 3 University of Kirkuk, Kirkuk, Kirkuk Governorate, Iraq

**Keywords:** Smart governance, digital transformation, sustainable development

## Abstract

**Background:**

With modern technology ever more entrenched in tertiary schooling, digital change has become an urgent necessity to enhance both the educational and administrative processes within universities. Anbar, Fallujah, and Maarif Universities now aim to adopt digital transformation strategies to improve academic and administrative efficiency while developing services provided to students and faculty. Within this framework, savvy governance plays a pivotal role in ensuring the application of principles like transparency, accountability, and participation, which are core factors for boosting the effectiveness of digital change. Literature indicates that integrating smart governance practices into higher education institutions contributes to achieving a modern and flexible educational environment with the dexterity to keep pace with technological progress.

**Methodology:**

The research followed a descriptive and analytical approach. The method used was a questionnaire administered to a sample of 83 employees in positions (dean, assistant dean, department head) at the universities under study (Anbar University, Fallujah University, and Al-Maarif University). The study will be conducted over the period from April 7, 2025, to September 1, 2025.

**Results:**

The descriptive results reveal that the university has consistently adopted smart governance tools, particularly the efficient use of the dimensions of transparency, participation, accountability, collaboration, and innovation, which have contributed to the universities’ transformation into a digital system. The study also demonstrated a high positive correlation of 0.93 between smart governance and digital transformation in the universities in the study area, confirming the close relationship between the studied variables.

**Conclusions:**

Integrating smart governance with digital transformation plays a pivotal role in enhancing institutional efficiency by promoting transparency, accountability, and innovation. This contributes to improving service quality, increasing beneficiary satisfaction, and supporting sustainable development.

## Introduction

Smart governance holds great potential for improving institutional effectiveness when implemented properly. It is built upon principles of transparency, accountability, participation, collaboration and innovation. As the independent variable of this study, smart governance can enhance administrative processes and decision making through strategic use of emerging technologies. Digital transformation, the dependent variable, entails fully integrating digital tools across operations, education services and strategies.

Previous research corroborates that smart governance bolsters efficiency and advances sustainable development goals. An ( Arnold et al., 2021) analysis highlighted how digitization supported continuity of learning and resilience amidst the COVID-19 pandemic. A ( Hai et al., 2021) paper explored opportunities and hurdles for leaders digitalizing in developing nations. ( Reis & Melão, 2023) proposed frameworks for holistically transitioning. And according to ( Ciarli et al., 2021) digital transformation in higher education merits attention due to implications for skills, innovation and competitiveness.

This research addresses gaps in knowledge pertaining to the smart governance-digital transformation relationship specific to Iraqi universities. The problem stems from needing to comprehend how smart governance dimensions impact success when implementing digital transformation and achieving performance targets. As such, the objective involves exploring impact, determining extent of influence on performance, and juxtaposing findings against other work to pinpoint areas for growth within the local setting.

Accordingly, the research hypotheses were formulated that there is a positive relationship between transparency, participation, and innovation in smart governance and digital transformation, and that digital transformation has a positive impact on institutional performance. This adds distinct value to the research by integrating the two variables in the context of Iraqi universities and from the perspective of university leaders, thus addressing the gap in foreign studies that focused on non-educational contexts or in countries with different environments.

## Theoretical framework

### First: Smart governance

Academic interest in the concept of smart governance began to increase at the beginning of the third millennium, as a natural development of previous stages of governance concepts that were primarily linked to information and communications technologies, most notably e-governance. E-governance has emerged as a means of improving the effectiveness and efficiency of government work through digitization. However, it has focused primarily on digital public services rather than reshaping the relationship between governance and citizens ( Sebastian, 2017: 101).

With the development of smart city concepts, thinking has begun to shift toward a new type of governance that leverages advanced digital technology not only in service provision, but also in redefining the relationship between state and society through innovation and open participation. This is called smart governance ( Tranos & Gertner, 2012: 177). Smart governance has become an essential part of smart city systems, seeking to integrate technology with democratic and administrative practices to enhance efficiency, transparency, accountability, and innovation.

Based on this, ( Barrionuevo et al., 2012: 152) indicated that smart cities need to develop smart governance systems that take into account all basic processes through three stages: beginning with understanding the situation, then diagnosing the situation, then developing a strategic plan, and finally taking appropriate action.

From this perspective, smart governance can be viewed as a set of principles, factors, and capabilities that constitute a form of governance capable of adapting to the complexities of a knowledge society ( Vial, 2019: 124).

( Gil-Garcı’a, 2012: 274) adds that smart governance is “the activity that coordinates communications to achieve collective goals through cooperation.”

The primary goal of smart governance is not limited to digitizing procedures alone, but rather extends to developing open interactive models between governments, citizens, and the private sector, leading to increased government efficiency, enhanced political legitimacy, and more effective responses to social and economic changes ( Vial, 2019: 123).

### Dimensions of smart governance

According to the analysis provided by ( Bolívar & Meijer, 2016: 4–6) smart governance is based on several key dimensions that represent the core of its functions and mechanisms. These can be summarized as follows:

1. Transparency: Smart governance seeks to make government information clearly available to citizens, enhancing trust and reducing opportunities for corruption, through the intelligent use of technology to reveal performance and policies ( Giffinger et al., 2007: 1).
2. Participation: Enabling citizens to actively participate in policy formulation and decision-making through interactive technological channels that allow them to directly influence governance outcomes ( Sebastian, 2017: 106).
3. Accountability: Activating technological tools that enable the monitoring of government administrative and financial performance and ensuring that officials are held accountable through open data and reports ( Vial, 2019: 123).
4. Collaborative Governance: Encouraging joint collaboration between governments, businesses, and citizens in policy development. Providing public solutions in a collaborative, collective manner, supported by digital technologies ( Barrionuevo, 2012: 52).
5. Innovation: Encouraging governments to adopt new and innovative technological solutions, allowing greater flexibility in addressing challenges and providing an environment conducive to experimentation and generating smart alternatives ( Odendaal, 2003: 586).

### Second: Digital transformation

While computation has influenced organizations since the 1960s, automating processes merely skimmed the surface of digital transformation’s potential. As technological capabilities expanded, a comprehensive revolution fundamentally reshaped how all sectors operate. Today, digital strategy represents the cornerstone for growth and adaptation over coming decades.

For businesses, digital transformation comprises a profound overhaul, incorporating infrastructure development, optimized technology use, refined communications, and reskilled employees to satisfy current and future needs. It strategically leverages modern digital tools to revolutionize models and workflows, enhancing efficiency, quality, and satisfaction. In banking, innovations like cloud, blockchain, AI, and e-banking automate internal functions while establishing digital platforms.

This transformation also supplies users with comprehensive, accurate data for informed economic choices, granting competitive advantages. Researchers view it as a mechanism facilitating nimbler reporting and bolstering resilience during disruptions. Meanwhile, others see it as reengineering income sources and value through reimagined models tailored to technology.

The importance stems from digital transformation’s pivotal influence across daily experiences, empowering organizations to evolve relentlessly, maximize talent, and foster sustainable innovation ensuring competitiveness. It further contributes to achieving global development targets and disseminating knowledge boundlessly across digital networks. ( Valdés et al., 2021: 5).

### Dimensions of digital transformation

( Matt et al., 2015:340) classified the dimensions of digital transformation into four main axes:

1. The technological dimension, which reflects the organization’s awareness of the vision and strategies for adopting technology, its use for innovation, data collection and analysis, and the preparation of accurate reports, as well as the creation of new job opportunities ( Ciarli et al., 2021:2).
2. The value creation dimension, which addresses the impact of digital transformation on operations compared to traditional methods, and the potential it offers for innovating knowledge-based products and services and enhancing competitive advantage ( Parviainen et al., 2017:74).
3. Structural changes, as digital transformation requires restructuring organizational activities or integrating them with the existing structure, in addition to aligning the organizational culture with the requirements of change ( Sabuncu, 2022:105).
4. The financial dimension, which is essential for providing the necessary funding for purchasing equipment, conducting maintenance, and implementing training programs, investments that can be expensive ( Özdemir, 2023:83).

### The relationship between smart governance and digital transformation

The relationship between the research variables and their dimensions is embodied in the functional interdependence between smart governance and the requirements of digital transformation. The application of smart governance principles, with their dimensions of transparency, participation, accountability, partnership, and innovation, creates a supportive environment for achieving digital transformation in public sector organizations. Transparency contributes to enhancing the flow of digital information and improving the quality of data used in transformation systems ( Giffinger et al., 2007:1), while community participation through interactive digital channels provides greater opportunities for designing integrated digital services ( Sebastian, 2017:106). Accountability enhances the reliability of digital systems by tracking performance and disclosing results using open data tools ( Vial, 2019:123). Partnership creates integration between the government, the private sector, and society, which increases the efficiency of the digital infrastructure ( Barrionuevo, 2012:52), while innovation represents the main driver for adopting smart solutions and modern technologies in transformation management ( Odendaal, 2003:586). On the other hand, digital transformation, with its technological, value creation, structural, and financial dimensions ( Matt et al., 2015:340), depends heavily on the effectiveness of smart governance practices. The application of transparency and accountability contributes to the efficient management of financial resources ( Özdemir, 2023:83), while innovation and participation enhance the technological dimension and process restructuring ( Sabuncu, 2022:105). Thus, the integration of smart governance and digital transformation represents a reciprocal relationship that leads to building a flexible administrative system capable of responding to contemporary changes and challenges ( Valdés et al., 2021:5).

## Methodology

### Type, design, and scope

This study adopted a descriptive-analytical approach, due to its ability to describe and analyze the phenomena under study. First, the research variables—smart governance in its dimensions (transparency, participation, accountability, participatory, Creativity) and digital transformation in its dimensions (technological dimension, value creation dimension, structural changes, financial dimension)—were described using a questionnaire designed for this purpose. The data were then statistically analyzed using appropriate statistical methods to extract indicators and interpret the relationships between the variables, allowing for a deeper understanding of the current reality of the Iraqi universities under study.

### Population and sample

The research community consists of all university leaders at the universities studied (University of Anbar, University of Fallujah, University of Maaref ), who hold the positions of (Dean, Assistant Dean, Head of Department), and a total of (83) employees. A comprehensive enumeration method was adopted in selecting the sample, so that the sample encompassed the entire research community. This ensures the inclusion of all statistical units relevant to the study topic and provides accurate data that reflects the actual situation.

### Procedure

According to the research design, the study followed logical and systematic steps, facilitating the solution of the research problem. The most important research steps included the following points: conceptualizing the idea, stating the problem, designing the research, selecting the sample, collecting data, analyzing and interpreting it, and discussing the results.

### Techniques and data collection instruments

Given the study’s dimensions and indicators, a questionnaire was adopted as the primary means of data collection. The questionnaire was validated in two consecutive stages to ensure the accuracy of the tool and its suitability for the study objectives. The first stage included content validity, where the questionnaire was presented to a committee of academic experts and specialists in the field to review the clarity, relevance, and comprehensiveness of its items. Based on their feedback, modifications were made to improve the wording and ensure its alignment with the study objectives. The second stage involved a pilot test on a small sample of the study population to verify the clarity of the questions and identify any potential ambiguities. The results confirmed the validity of the questionnaire for use in research. This tool was developed to include the smart governance variable, consisting of ( Jameel, 2022) items distributed across its dimensions, using a five-point Likert scale to determine the level of response. The digital transformation variable included ( Hai et al., 2021) items distributed across its dimensions, using the same scale. The questionnaire was administered to the study sample to obtain the data necessary for statistical analysis.

### Ethical considerations of the study

The researcher was keen to observe ethical considerations during all stages of the study, from data collection to presentation of results. These considerations included informing the sample members of the nature and objectives of the study before participating, and ensuring that their participation was entirely voluntary and without any pressure. The confidentiality of the information obtained was emphasized and that it would be used only for scientific research purposes. No personal data that could reveal the identity of the participants was included. The researcher also adhered to complete objectivity and neutrality throughout all stages of data collection, analysis, and presentation of results, avoiding any distortion or selection of information to ensure scientific integrity.

### Analysis of results

The study aimed to determine the relationship and impact between smart governance and digital transformation for a sample of leaders at selected Iraqi universities. The study was conducted based on questionnaire data, and according to the study variables, the following was observed:

1. **Description of Respondents**

This paragraph will describe the general information of the respondents, as shown in Table 1 below:

**Table 1.** Description of the researched individuals.

Description	Division or category	number	ratio
Type	male	69	83%
	feminine	14	17%
	total	83	100%
the age	Under 30	45	54.2%
	30–35 years	19	22.9%
	36–45 years	13	15.7%
	46–55 years	3	3.6%
	Over 55	3	3.6%
	total	83	100%
Job title	Dean	7	8.4%
	Assistant Dean	18	21.7%
	Head of Department	58	69.9%
	Total	83	100%
Academic qualification	Master’s	63	75.9%
	PhD	20	24.1%
	Total	83	100%
years of experience	Less than 5 years	41	49.4%
	( Campbell & Flux, 1952; Ciarli et al., 2021; Development, R, 1995; Galliers & Jarvenpaa, 2010; Giffinger et al., 2007; Gil-Garcia, 2015)	23	27.7%
	(More than 10)	19	22.9%
	Total	83	100%

Gender: Males accounted for 83% of respondents, while females accounted for 17%.

Age: The age group (under 30) accounted for 54.2%, while the age group (30–35) accounted for 22.9%, the age group (36–40) accounted for 15.7%, the age group (46–55) accounted for 3.6%, and the age group (over 55) accounted for 3.6% of respondents.

Job Title: The job title “Dean” accounted for 8.4% of respondents, while the job title “Assistant Dean” accounted for 21.7%, and the job title “Head of Department” accounted for 69.9% of respondents.

Academic Qualification: The percentage of those with a Master’s degree was 75.9%, while the percentage of those with a PhD was 24.1%.

Years of experience: It is noted that years of experience (less than 5 years) accounted for 49.4%, while years of experience ( Campbell & Flux, 1952; Ciarli et al., 2021; Development, R, 1995; Galliers & Jarvenpaa, 2010; Giffinger et al., 2007; Gil-Garcia, 2015) years accounted for 27.7%, and finally, years of experience (more than 10) years accounted for 22.9% of respondents.

2. **Diagnosis and Description of Study Variables**

Describing and diagnosing study variables at the aggregate level and in terms of their dimensions is an important step in determining the level of orientation and inclination of the research subjects toward these variables and dimensions. These inclinations are usually measured through the percentage of agreement and the relative importance index (RII), This index is used to determine the relative importance of the study’s items and dimensions from the perspective of the research sample in the organization being studied. Note that the value of this index falls between (0 ≤ RII ≤ 1) and can be classified into five levels according to the five-point Likert scale adopted in our study, as shown in Table 2.

**Table 2.** Importance levels according to the five-point Likert scale.

relative importance	RII
High	0.8 ≤ RII ≤ 1
Medium to High	0.6 ≤ RII < 0.8
Medium	0.4 ≤ RII < 0.6
Low to Medium	0.2 ≤ RII < 0.4
Low	0 ≤ RII < 0.2

**Description and diagnosis of the independent variable, smart governance**

This section will address the dimensions of the smart governance variable and the results shown in Table 3 below:

**Table 3.** Dimensions of the smart governance variable and the results shown in it.

sequence	Dimensions	arithmetic mean	standard deviation	Response level%
1	Transparency	3.574	1.011	71.486
2	Participation	3.755	0.866	75.101
3	Accountability	3.799	0.833	75.984
4	Participatory	3.900	0.745	77.992
5	Creativity	3.353	0.851	67.068

This paragraph will describe the dimensions of the smart governance variable and the results in Table 3 below:

The average of this dimension of transparency was (3.574) with a standard deviation of (1.011) and a response intensity of (71.486%), which is classified as a medium to high percentage as an indicator of relative importance (RII). The average of the participation dimension was (3.755) with a standard deviation of (0.866) and a response intensity of (75.101%), which is classified as a medium to high percentage as an indicator of relative importance (RII). The average of the accountability dimension was (3.799) with a standard deviation of (0.833) and a response intensity of (75.984%), which is classified as a medium to high percentage as an indicator of relative importance (RII). The average of the participatory dimension was (3.90) with a standard deviation of (0.745) and a response intensity of (77.992%), which is classified as a medium to high percentage as an indicator of relative importance (RII). The average of the innovation dimension was (3.353) with a standard deviation of (0.851) and a response intensity of (67.068%), which is classified as a medium to high percentage as an indicator of relative importance (RII).

**Description and diagnosis of the dependent variable: digital transformation**

This paragraph will describe the dimensions of the digital transformation variable and the results in Table 4 below:

**Table 4.** Dimensions of the digital transformation variable and results.

sequence	Dimensions	arithmetic mean	standard deviation	Response level%
1	technological dimension	4.032	0.889	80.643
2	creating value	3.663	1.050	73.253
3	structural changes	3.847	0.895	76.947
4	Financial dimension	4.088	0.777	81.767

The average of the technology dimension was (4.032) with a standard deviation of (0.889) and a response intensity of (80.643%), which is classified as a high percentage as a Relative Importance Index (RII). The average of the value creation dimension was (3.663) with a standard deviation of (1.050) and a response intensity of (73.253%), which is classified as a medium to high percentage as a Relative Importance Index (RII). The average of the structural changes dimension was (3.847) with a standard deviation of (0.895) and a response intensity of (76.947%), which is classified as a medium to high percentage as a Relative Importance Index (RII). The average of the financial dimension was (4.088) with a standard deviation of (0.777) and a response intensity of (81.767%), which is classified as a high percentage as a Relative Importance Index (RII).

**Relative importance**

Table 5 shows the relative importance of the two variables (smart governance and digital transformation) from the responses of a sample in selected Iraqi universities, as follows:

**Table 5.** Relative importance of the dimensions of the study variables.

sequence	Variables	arithmetic mean	standard deviation	Response level%	Arrangement
1	Smart Governance	3.676	0.861	73.526	2
2	Digital Transformation	3.908	0.903	78.153	1

From Table 5 above, Digital transformation achieved a relatively higher edge (78.153%) over smart governance (73.526%), basically demonstrating the more important variable among the participants.

3. **Correlation and influence analysis of study variables**

**Correlation analysis**

The correlation coefficient is a measure that determines the strength and type of relationship between two variables. The correlation coefficient indicates the type of relationship between the variables, whether positive or negative. The correlation coefficient value also represents the strength of the relationship between them. The closer the correlation coefficient value is to one, whether positive or negative, the stronger the relationship between the two variables. Furthermore, the correlation coefficient may be significant or insignificant, which is determined by the P value. If the P value is less than 0.05, this indicates that the correlation coefficient is significant, and vice versa. Table 6 below shows the results of the correlation test between the study variables:

**Table 6.** Correlation between the smart governance variable and the digital transformation variable.

independent variable	Relationship direction	dependent variable	Link value	Confidence Interval 95%		P-value
				Lower	Upper	
Smart Governance	↔	Digital transformation	0.93	0.851	0.987	0.003

**Figure 1.**
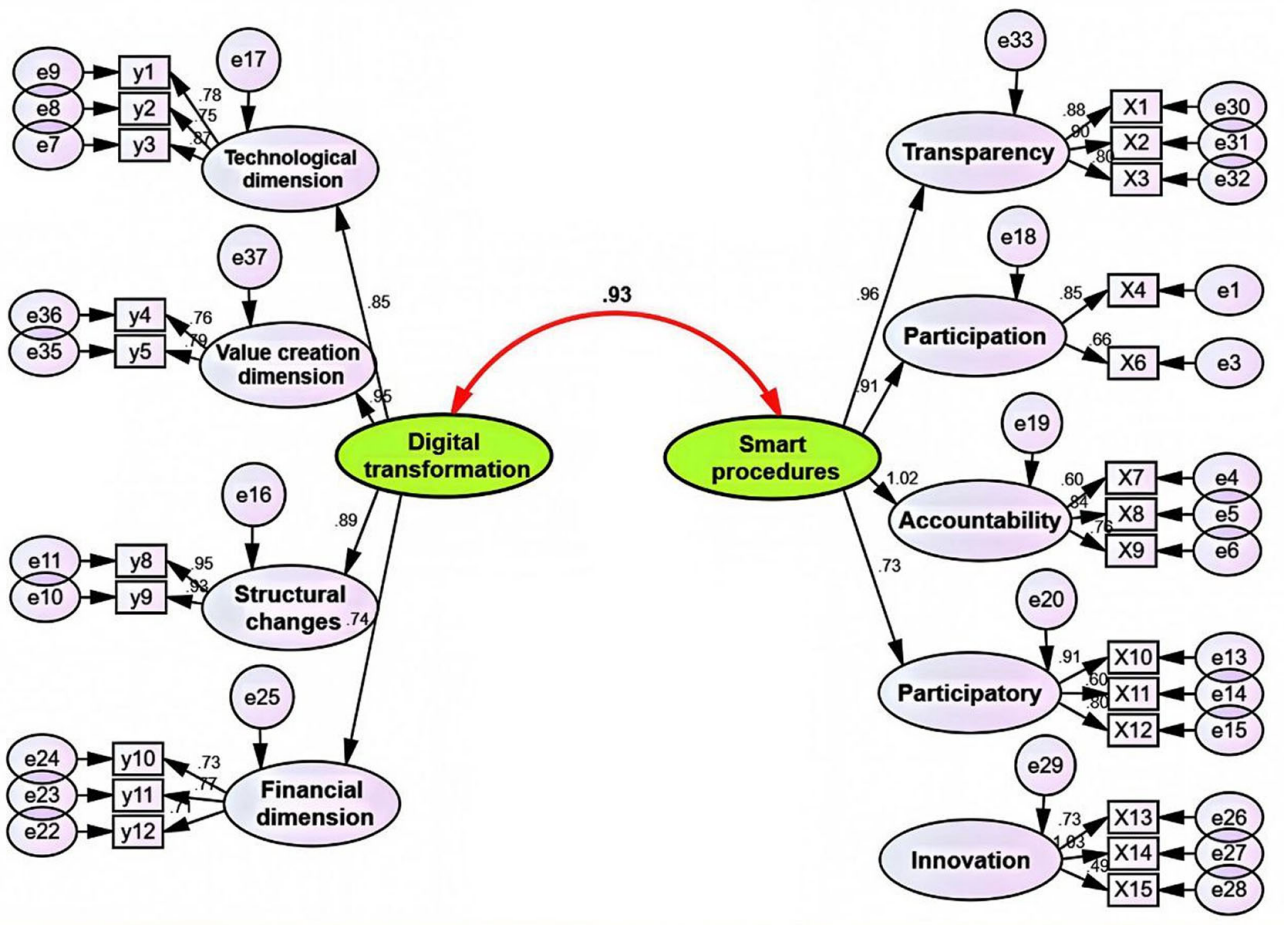
The correlation between the smart governance variable and the digital transformation variable.

The correlation between the smart governance and the digital transformation is a direct relationship, as evidenced by the strong positive correlation coefficient value, which reached (0.93). The confidence interval (0.851 to 0.987), which translate to 95% and a Pvalue of 0.003 (<0.05) consolidate the statistical significance of this relationship. The correlation between the smart governance variable and the digital transformation variable can be observed in Figure 1:

**Impact analysis between study variables**. Table 7 below shows the results of the impact test between study variables.

**Table 7.** Regression Analysis of the Impact Between Variables.

independent variable	Direction of influence	dependent variable	Estimate(β)	Se.(β)	Confidence Interval 95%		P-value
					Lower	Upper	
Smart Governance	→	Digital transformation	1.389	0.529	2.789	0.741	0.002

**Figure 2.**
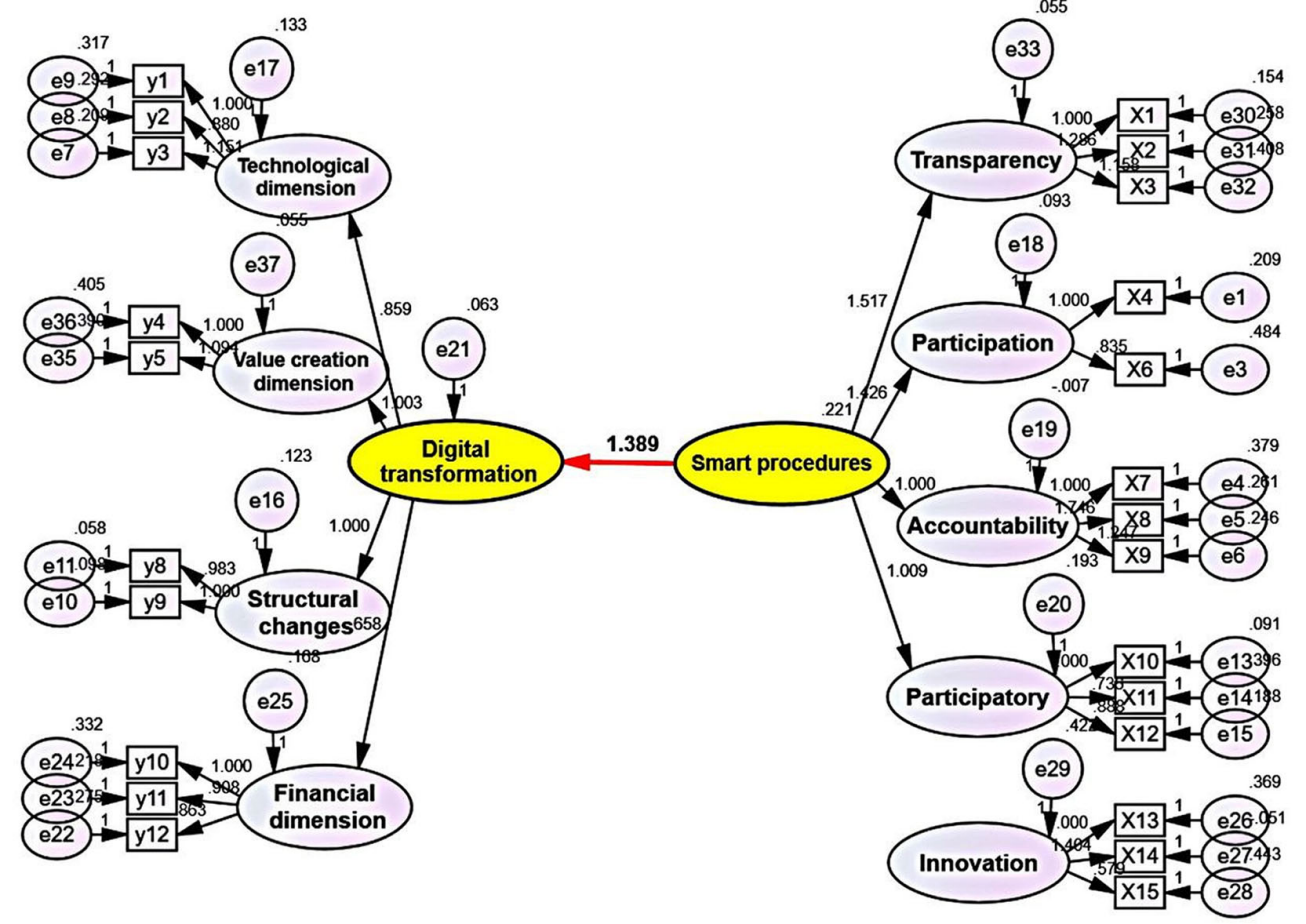
The impact relationship of the smart governance variable on the digital transformation variable.

The impact of the smart governance variable on the digital transformation variable is demonstrated by the estimated parameter attributed to the smart governance variable, which amounted to (1.389), indicating a direct relationship between the smart governance variable and the digital transformation variable, given the positive sign of this estimated parameter. The standard error value (S.E.) amounted to (0.529). NOTE: CONSIDER REWRITING THE YELLOW SECTION: The analysis signifies that smart governance, with an estimated parameter of (1.389), bears an influential significance on digital transformation. Furthermore, the impact of the smart governance variable on the digital transformation variable was statistically significant, as the p-value (0.002) appeared less than (0.05) for this relationship. The confidence interval (95%) appeared with similar signs, represented by the minimum and maximum limits (2.789 and 0.741), respectively. Therefore, based on the above results, a decision can be reached to accept the alternative hypothesis stating that the smart governance variable has an impact on the digital transformation variable. The impact of the smart governance variable on the digital transformation variable can be observed in Figure 2:

## Discussion - conclusions – recommendations

### Discussion

The results showed that the level of smart governance in the surveyed universities ranged from medium to high, reflecting a noticeable trend toward adopting its core principles of transparency, participation, accountability, and collaboration. Meanwhile, the innovation dimension emerged at a relatively lower level, indicating that universities still need to enhance their ability to embrace innovative ideas and develop new mechanisms to support this aspect. Regarding digital transformation, the results showed that universities are making clear progress, particularly in technological infrastructure and the financial aspect, reflecting serious investment in digital resources and technologies. Meanwhile, the dimensions of value creation and structural change were at good levels but require further support. Correlation analysis also revealed a strong, direct relationship between smart governance and digital transformation, indicating that strengthening governance principles directly contributes to accelerating and facilitating the digital transformation process. Impact analysis confirmed that smart governance is a key factor in achieving digital transformation requirements, consistent with previous literature and studies that have indicated that the success of digital projects depends largely on the presence of sound and effective governance systems.

### Conclusions

Indeed, universities in Iraq have notably evolved in their journeys towards intelligent administration and digital change. While governance remains devoted to its fundamental principles, innovation has become a crucial pillar necessitating promotion. On the contrary, digital transformation is progressing rapidly regarding technology and finances, cultivating fertile ground to hasten institutional progress. The conspicuous correlation and impact between intelligent administration and digital change confirms adopting governance principles is not merely an optional management decision, but rather a strategic necessity guaranteeing digital change’s success and sustainability within universities. A complex web of interactions between smart governance and digital transformation has formed, each reinforcing and necessitating the other in an effort to optimize operations in a modern technological landscape.

### Recommendations

Universities hold the key to shaping innovation through prudent administration. By inspiring novel ideas and embracing emerging technologies, together with engaging stakeholders in crucial discussions, digital change can be steered fruitfully. Continued funding of technological infrastructure requires transparency to optimize limited means. Foremost, an overarching vision is needed—one connecting leadership fundamentals with transformation necessities, balancing progress responsibly. Collaborations within and beyond university walls likewise let experience be shared and the forefront of practice observed globally.

## Ethical considerations

This study involved human participants and was conducted in accordance with accepted ethical research standards and the principles outlined in the Declaration of Helsinki. Ethical approval was obtained from the Scientific Research Ethics Committee, University of Fallujah, Iraq (Approval No. HOF.HUM.2025.001). Written informed consent was obtained from all participants prior to their participation. All participants were informed about the purpose of the study, the voluntary nature of their participation, their right to withdraw at any time without consequences, and the confidentiality of their data.

## Informed consent

Participation in this study was voluntary. Before completing the questionnaire, participants were provided with a brief explanation of the study objectives and were informed that their responses would remain anonymous and confidential. By completing and submitting the questionnaire, participants indicated their informed consent to participate in the study.

## Data Availability

Repository name: Zenodo: Smart governance and its role in achieving digital transformation requirements: A survey study in selected Iraqi universities. https://doi.org/10.5281/zenodo.19019843 [ Ghareeb, et al. 2026]. The project contains the following underlying data:
• Data.xlsx (Raw data used for statistical analysis). • SPSS.sav (Dataset used for statistical analysis in SPSS). Repository name: Zenodo: Smart governance and its role in achieving digital transformation requirements: A survey study in selected Iraqi universities. https://doi.org/10.5281/zenodo.19019843 [ Ghareeb, et al. 2026]. This project contains the following extended data:
• Questionnaire.pdf (Survey instrument used for data collection). Data are available under the terms of the Creative Commons Zero “No rights reserved” data waiver (CC0 1.0 Public domain dedication). This study is an observational survey-based research and follows the STROBE reporting guidelines. No CONSORT or ARRIVE checklists are required, as the study does not involve clinical trials or animal experiments.
