# Smart governance and its role in achieving digital transformation requirements: A survey study in selected Iraqi universities

**DOI:** 10.12688/f1000research.174187.2

**Published:** 2026-10-05

**Authors:** Ahmed Majeed Ghareeb, Muhamed Khamis Sami Alisawi, Yousif Obed Hama Amin, Younis M. Kh. Al-Sabaawe, Ahmed Abbas Hammadi, Sara Hamid diab

**Affiliations:** 1 University of Fallujah, Al-Fallujah, Al Anbar Governorate, Iraq; 2 University of Human Development, Sulaymaniyah, Kurdistan Region, Iraq; 3 University of Kirkuk, Kirkuk, Kirkuk Governorate, Iraq

**Keywords:** Smart governance, digital transformation, sustainable development

## Abstract

**Background:**

With modern technology ever more entrenched in tertiary schooling, digital change has become an urgent necessity to enhance both the educational and administrative processes within universities. Anbar, Fallujah, and Maarif Universities now aim to adopt digital transformation strategies to improve academic and administrative efficiency while developing services provided to students and faculty. Within this framework, savvy governance plays a pivotal role in ensuring the application of principles like transparency, accountability, and participation, which are core factors for boosting the effectiveness of digital change. Literature indicates that integrating smart governance practices into higher education institutions contributes to achieving a modern and flexible educational environment with the dexterity to keep pace with technological progress.

**Methodology:**

The research followed a descriptive and analytical approach. The method used was a questionnaire administered to a sample of 83 employees in positions (dean, assistant dean, department head) at the universities under study (Anbar University, Fallujah University, and Al-Maarif University). The study will be conducted over the period from April 7, 2025, to September 1, 2025.

**Results:**

The descriptive results reveal that the university has consistently adopted smart governance tools, particularly the efficient use of the dimensions of transparency, participation, accountability, collaboration, and innovation, which have contributed to the universities’ transformation into a digital system. The study also demonstrated a high positive correlation of 0.93 between smart governance and digital transformation in the universities in the study area, confirming the close relationship between the studied variables.

**Conclusions:**

Integrating smart governance with digital transformation plays a pivotal role in enhancing institutional efficiency by promoting transparency, accountability, and innovation. This contributes to improving service quality, increasing beneficiary satisfaction, and supporting sustainable development.

## Introduction

Smart governance holds great potential for improving institutional effectiveness when implemented properly. It is built upon principles of transparency, accountability, participation, collaboration and innovation. As the independent variable of this study, smart governance can enhance administrative processes and decision making through strategic use of emerging technologies. Digital transformation, the dependent variable, entails fully integrating digital tools across operations, education services and strategies.

Previous research corroborates that smart governance bolsters efficiency and advances sustainable development goals. An ( Arnold et al., 2021) analysis highlighted how digitization supported continuity of learning and resilience amidst the COVID-19 pandemic. A ( Hai et al., 2021) paper explored opportunities and hurdles for leaders digitalizing in developing nations. ( Reis & Melão, 2023) proposed frameworks for holistically transitioning. And according to ( Ciarli et al., 2021) digital transformation in higher education merits attention due to implications for skills, innovation and competitiveness.

This research addresses gaps in knowledge pertaining to the smart governance-digital transformation relationship specific to Iraqi universities. The problem stems from needing to comprehend how smart governance dimensions impact success when implementing digital transformation and achieving performance targets. As such, the objective involves exploring impact, determining extent of influence on performance, and juxtaposing findings against other work to pinpoint areas for growth within the local setting.

Accordingly, the research hypotheses were formulated that there is a positive relationship between transparency, participation, and innovation in smart governance and digital transformation, and that digital transformation has a positive impact on institutional performance. This adds distinct value to the research by integrating the two variables in the context of Iraqi universities and from the perspective of university leaders, thus addressing the gap in foreign studies that focused on non-educational contexts or in countries with different environments.

**Accordingly, the research aims to:**

1. Examine the level of smart governance practices in selected Iraqi universities.
2. Determine the level of digital transformation requirements in the universities included in the study.
3. Analyze the relationship between smart governance and digital transformation.
4. Determine the impact of smart governance dimensions on achieving the requirements of digital transformation in Iraqi universities.

## Theoretical framework

### First: Smart governance

Academic interest in the concept of smart governance began to increase at the beginning of the third millennium, as a natural development of previous stages of governance concepts that were primarily linked to information and communications technologies, most notably e-governance. E-governance has emerged as a means of improving the effectiveness and efficiency of government work through digitization. However, it has focused primarily on digital public services rather than reshaping the relationship between governance and citizens ( Sebastian, 2017).

With the development of smart city concepts, thinking has begun to shift toward a new type of governance that leverages advanced digital technology not only in service provision, but also in redefining the relationship between state and society through innovation and open participation. This is called smart governance ( Tranos & Gertner, 2012). Smart governance has become an essential part of smart city systems, seeking to integrate technology with democratic and administrative practices to enhance efficiency, transparency, accountability, and creativity.

Recent studies indicate that smart governance relies on digital readiness and innovative leadership to improve governance effectiveness and public service quality ( Aldhi et al., 2025).

Based on this, ( Barrionuevo et al., 2012) indicated that smart cities need to develop smart governance systems that take into account all basic processes through three stages: beginning with understanding the situation, then diagnosing the situation, then developing a strategic plan, and finally taking appropriate action.

From this perspective, smart governance can be viewed as a set of principles, factors, and capabilities that constitute a form of governance capable of adapting to the complexities of a knowledge society ( Vial, 2019).

( Gil-Garcia, 2012) adds that smart governance is “the activity that coordinates communications to achieve collective goals through cooperation.”

The primary goal of smart governance is not limited to digitizing procedures alone, but rather extends to developing open interactive models between governments, citizens, and the private sector, leading to increased government efficiency, enhanced political legitimacy, and more effective responses to social and economic changes ( Vial, 2019).

### Dimensions of smart governance

According to the analysis provided by ( Bolívar & Meijer, 2016) smart governance is based on several key dimensions that represent the core of its functions and mechanisms. These can be summarized as follows:

1. Transparency: Smart governance seeks to make government information clearly available to citizens, enhancing trust and reducing opportunities for corruption, through the intelligent use of technology to reveal performance and policies ( Giffinger et al., 2007).
2. Participation: Enabling citizens to actively participate in policy formulation and decision-making through interactive technological channels that allow them to directly influence governance outcomes ( Sebastian, 2017).
3. Accountability: Activating technological tools that enable the monitoring of government administrative and financial performance and ensuring that officials are held accountable through open data and reports ( Vial, 2019).
4. Collaborative Governance: Encouraging joint collaboration between governments, businesses, and citizens in policy development. Providing public solutions in a collaborative, collective manner, supported by digital technologies ( Barrionuevo, 2012).
5. Innovation: Encouraging governments to adopt new and innovative technological solutions, allowing greater flexibility in addressing challenges and providing an environment conducive to experimentation and generating smart alternatives ( Odendaal, 2003).

### Second: Digital transformation

While computation has influenced organizations since the 1960s, automating processes merely skimmed the surface of digital transformation’s potential. As technological capabilities expanded, a comprehensive revolution fundamentally reshaped how all sectors operate. Today, digital strategy represents the cornerstone for growth and adaptation over coming decades. Digital transformation increasingly depends on AI, IoT, and data-driven systems to improve efficiency and support smart decision-making ( Lloret et al., 2025).

For businesses, digital transformation comprises a profound overhaul, incorporating infrastructure development, optimized technology use, refined communications, and reskilled employees to satisfy current and future needs. It strategically leverages modern digital tools to revolutionize models and workflows, enhancing efficiency, quality, and satisfaction. In banking, innovations like cloud, blockchain, AI, and e-banking automate internal functions while establishing digital platforms.

This transformation also supplies users with comprehensive, accurate data for informed economic choices, granting competitive advantages. Researchers view it as a mechanism facilitating nimbler reporting and bolstering resilience during disruptions. Meanwhile, others see it as reengineering income sources and value through reimagined models tailored to technology. Digital transformation contributes to creating public value through better information sharing and more sustainable digital policies ( Lee & Yoon, 2024).

The importance stems from digital transformation’s pivotal influence across daily experiences, empowering organizations to evolve relentlessly, maximize talent, and foster sustainable innovation ensuring competitiveness. It further contributes to achieving global development targets and disseminating knowledge boundlessly across digital networks. ( Valdés et al., 2021).

### Dimensions of digital transformation

( Matt et al., 2015) classified the dimensions of digital transformation into four main axes:

1. The technological dimension, which reflects the organization’s awareness of the vision and strategies for adopting technology, its use for innovation, data collection and analysis, and the preparation of accurate reports, as well as the creation of new job opportunities ( Ciarli et al., 2021).
2. The value creation dimension, which addresses the impact of digital transformation on operations compared to traditional methods, and the potential it offers for innovating knowledge-based products and services and enhancing competitive advantage ( Parviainen et al., 2017).
3. Structural changes, as digital transformation requires restructuring organizational activities or integrating them with the existing structure, in addition to aligning the organizational culture with the requirements of change ( Sabuncu, 2022).
4. The financial dimension, which is essential for providing the necessary funding for purchasing equipment, conducting maintenance, and implementing training programs, investments that can be expensive ( Özdemir, 2023).

### The relationship between smart governance and digital transformation

The relationship between the research variables and their dimensions is embodied in the functional interdependence between smart governance and the requirements of digital transformation. The application of smart governance principles, with their dimensions of transparency, participation, accountability, partnership, and innovation, creates a supportive environment for achieving digital transformation in public sector organizations. Transparency contributes to enhancing the flow of digital information and improving the quality of data used in transformation systems ( Giffinger et al., 2007), while community participation through interactive digital channels provides greater opportunities for designing integrated digital services ( Sebastian, 2017). Accountability enhances the reliability of digital systems by tracking performance and disclosing results using open data tools ( Vial, 2019). Partnership creates integration between the government, the private sector, and society, which increases the efficiency of the digital infrastructure ( Barrionuevo et al., 2012), while innovation represents the main driver for adopting smart solutions and modern technologies in transformation management ( Odendaal, 2003). On the other hand, digital transformation, with its technological, value creation, structural, and financial dimensions ( Matt et al., 2015), depends heavily on the effectiveness of smart governance practices. The application of transparency and accountability contributes to the efficient management of financial resources ( Özdemir, 2023), while innovation and participation enhance the technological dimension and process restructuring ( Sabuncu, 2022). Thus, the integration of smart governance and digital transformation represents a reciprocal relationship that leads to building a flexible administrative system capable of responding to contemporary changes and challenges ( Valdés et al., 2021).

### Cross-referencing of previous studies

The literature shows a consistent relationship of smart governance with digital transformation in various areas. Research on digital transformation has focused on the fact that it is not just a technology solution, but a transformational change in the organisation. All of those ( Matt et al. 2015), ( Hanelt et al. 2021), and ( Reis & Melão, 2023) point to the need for strategic alignment, organizational readiness, and the creation of integrated digital strategies for successful digital transformation.

In the higher education and public sector, ( Valdés et al., 2021), ( Arnold et al., 2021), and ( Rampelt et al., 2019) have found that institutional structures and governance mechanisms are important factors in influencing outcomes of digital transformation. Likewise, ( Bolívar & Meijer, 2016) and ( Odendaal, 2003) highlight the key role of smart governance for transparency, participation, and efficiency of institutions, which are crucial for smart digital transformation of public organizations and smart environments.

Furthermore, ( Ciarli et al., 2021), and ( Hai et al., 2021) highlight the significance of human capital, leadership competences, and digital skills in supporting the transformation process, highlighting that technological infrastructures are not enough if they are not complemented by appropriate governance and capacity development mechanisms.

In terms of function and application, ( Jameel, 2022), and ( Qushtom, 2021) have all shown that digitized reporting systems and technologies, like XBRL, enhance the quality of information, transparency and the efficiency of decision making, which re-emphasises the governance role in the digital transformation processes.

Overall, the reviewed studies collectively indicate that smart governance serves as an enabling framework that integrates technological, organizational, and human dimensions, thereby ensuring the effectiveness and sustainability of digital transformation in different institutional settings.

### The role of theories in supporting the study

The stakeholder theory and resource theory are used to explain the relationship between smart governance and digital transformation in this study. The stakeholder theory is a concept that explains how to involve relevant stakeholders in the development of transparency, participation and accountability pillars of smart governance that support the success of digital transformation initiatives in HEIs.

Additionally, resource theory shows that an effective use of material, human and knowledge resources helps to increase the capacity of institutions to embrace digital transformation. Smart government aims to enhance the governance of such resources and their effective use.

Therefore, these two theories have a holistic explanation of the relationship between the research variables both behavioural and organizational and technological aspects.

### Research methodology and design

This research used quantitative approach to research the relationship between smart governance and the needs of digital transformation in selected Iraqi universities. Descriptive analytical research design was used which was considered appropriate for the description of the research variables, analysis of the relationships and interactions between the variables.

The research was conducted using primary and secondary sources of data. Primary data were gathered from a structured questionnaire sent to 83 university leaders (deans, assistant deans, and department heads) of the universities studied (University of Al-Maarif, University of Fallujah, and University of Anbar). Secondary data were collected from the various studies, journals, books, and researches on the concepts of smart governance and digital transformation.

The research was conducted from April 7, 2025, to September 1, 2025.

### Population and sample

The research community includes all the University leaders at the universities studied (University of Anbar, University of Fallujah, University of Maaref) and (Dean, Assistant Dean, Head of Department) in addition to a total of (83) employees. A comprehensive enumeration method was used in the selection of the sample, which means that all the community of the research was included in the sample. This guarantees that all statistical units that are relevant to the research subject are included in the research to get accurate data that reflects the reality.

### Procedure

The research design is logical and systematic as per the research problem and helps in deducing the solution. The most crucial research steps that were highlighted were as follows: conceptualization of the idea, problem statement, design of research, selection of sample, data collection, data analysis and data interpretation, and discussions of results.

### Techniques and data collection instruments

Because of the size of the research and the indicators, questionnaire was used as the tool for data collection. The questionnaire was validated twice in a row to ensure the accuracy of the tool and its suitability for the objectives of the study. The first phase was content validity, in which the questionnaire was shown to a panel of academic experts and specialists in the field for comment on the clarity, relevance and comprehensiveness of items. Suggestions from them were incorporated and changes were made to improve the wording and to make it match with the aims of the study. The second step was to conduct a pilot test with a small sample of the research population to ensure the questions were clear and easy to understand and to see if there were any ambiguities. The results confirmed the validity of the questionnaire for use in research. The tool was designed to add the variable of smart governance with (15) items divided into dimensions and then to use five-point Likert scale to decide on the level of the response. The digital transformation variable comprised 12 items across its dimensions, with the same scale. The respondents were given the questionnaire to collect the data for the statistical analysis.

### Ethical considerations of the study

The research was very interested in observing the ethical aspects throughout the research from data collection to presentation of the results. Some of these considerations were informing the sample members of the nature and purpose of the research prior to their participation, and allowing them to freely and without pressure participate in the study. Confidentiality of the information obtained was stressed and it would be used for scientific research only. There was no personal data that would allow the identification of the participants. The researcher also conducted himself/herself objectively and neutrally in all phases of the research process from the collection of data, analysis of data to the presentation of the results of the research, without distorting or selecting the information to achieve scientific integrity.

### Analysis of results

The research was designed to find out the relationship and impact between smart governance and digital transformation with regard to the sample of the leaders in the selected Iraqi universities. Based on the data obtained from the questionnaires, the following was observed:

1. **Description of Respondents**

This paragraph will describe the general information of the respondents, as shown in Table 1 below:

**Table 1.** Description of the researched individuals.

Description	Division or category	Number	Ratio
Type	Male	69	83%
	Feminine	14	17%
	Total	83	100%
The age	Under 30	45	54.2%
	30–35 years	19	22.9%
	36–45 years	13	15.7%
	46–55 years	3	3.6%
	Over 55	3	3.6%
	Total	83	100%
Job title	Dean	7	8.4%
	Assistant Dean	18	21.7%
	Head of Department	58	69.9%
	Total	83	100%
Academic qualification	Master’s	63	75.9%
	PhD	20	24.1%
	Total	83	100%
Years of experience	Less than 5 years	41	49.4%
	( Campbell & Flux, 1952; Ciarli et al., 2021; Development, R, 1995; Galliers & Jarvenpaa, 2010; Giffinger et al., 2007; Gil-Garcia, 2015)	23	27.7%
	(More than 10)	19	22.9%
	Total	83	100%

Gender: Males accounted for 83% of respondents, while females accounted for 17%.

Age: The age group (under 30) accounted for 54.2%, while the age group (30–35) accounted for 22.9%, the age group (36–40) accounted for 15.7%, the age group (46–55) accounted for 3.6%, and the age group (over 55) accounted for 3.6% of respondents.

Job Title: The job title “Dean” accounted for 8.4% of respondents, while the job title “Assistant Dean” accounted for 21.7%, and the job title “Head of Department” accounted for 69.9% of respondents.

Academic Qualification: The percentage of those with a Master’s degree was 75.9%, while the percentage of those with a PhD was 24.1%.

Years of experience: It is noted that years of experience (less than 5 years) accounted for 49.4%, while years of experience ( Campbell & Flux, 1952; Ciarli et al., 2021; Development, R, 1995; Galliers & Jarvenpaa, 2010; Giffinger et al., 2007; Gil-Garcia, 2015) years accounted for 27.7%, and finally, years of experience (more than 10) years accounted for 22.9% of respondents.

2. **Diagnosis and Description of research Variables**

Describing and diagnosing research variables at the aggregate level and in terms of their dimensions is an important step in determining the level of orientation and inclination of the research subjects toward these variables and dimensions. These inclinations are usually measured through the percentage of agreement and the relative importance index (RII), This index is used to determine the relative importance of the study’s items and dimensions from the perspective of the research sample in the organization being studied. Note that the value of this index falls between (0 ≤ RII ≤ 1) and can be classified into five levels according to the five-point Likert scale adopted in our study, as shown in Table 2.

**Table 2.** Importance levels according to the five-point Likert scale.

Relative importance	RII
High	0.8 ≤ RII ≤ 1
Medium to High	0.6 ≤ RII < 0.8
Medium	0.4 ≤ RII < 0.6
Low to Medium	0.2 ≤ RII < 0.4
Low	0 ≤ RII < 0.2

**Description and diagnosis of the independent variable, smart governance**

This section will address the dimensions of the smart governance variable and the results shown in Table 3 below:

**Table 3.** Dimensions of the smart governance variable and the results shown in it.

Sequence	Dimensions	Arithmetic mean	Standard deviation	Response level%
1	Transparency	3.574	1.011	71.486
2	Participation	3.755	0.866	75.101
3	Accountability	3.799	0.833	75.984
4	Participatory	3.900	0.745	77.992
5	Creativity	3.353	0.851	67.068

This paragraph will describe the dimensions of the smart governance variable and the results in Table 3 below:

The average of this dimension of transparency was (3.574) with a standard deviation of (1.011) and a response intensity of (71.486%), which is classified as a medium to high percentage as an indicator of relative importance (RII). The average of the participation dimension was (3.755) with a standard deviation of (0.866) and a response intensity of (75.101%), which is classified as a medium to high percentage as an indicator of relative importance (RII). The average of the accountability dimension was (3.799) with a standard deviation of (0.833) and a response intensity of (75.984%), which is classified as a medium to high percentage as an indicator of relative importance (RII). The average of the participatory dimension was (3.90) with a standard deviation of (0.745) and a response intensity of (77.992%), which is classified as a medium to high percentage as an indicator of relative importance (RII). The average of the innovation dimension was (3.353) with a standard deviation of (0.851) and a response intensity of (67.068%), which is classified as a medium to high percentage as an indicator of relative importance (RII).

**Description and diagnosis of the dependent variable: digital transformation**

This paragraph will describe the dimensions of the digital transformation variable and the results in Table 4 below:

**Table 4.** Dimensions of the digital transformation variable and results.

Sequence	Dimensions	Arithmetic mean	Standard deviation	Response level%
1	Technological dimension	4.032	0.889	80.643
2	Creating value	3.663	1.050	73.253
3	Structural changes	3.847	0.895	76.947
4	Financial dimension	4.088	0.777	81.767

The average of the technology dimension was (4.032) with a standard deviation of (0.889) and a response intensity of (80.643%), which is classified as a high percentage as a Relative Importance Index (RII). The average of the value creation dimension was (3.663) with a standard deviation of (1.050) and a response intensity of (73.253%), which is classified as a medium to high percentage as a Relative Importance Index (RII). The average of the structural changes dimension was (3.847) with a standard deviation of (0.895) and a response intensity of (76.947%), which is classified as a medium to high percentage as a Relative Importance Index (RII). The average of the financial dimension was (4.088) with a standard deviation of (0.777) and a response intensity of (81.767%), which is classified as a high percentage as a Relative Importance Index (RII).

**Relative importance**

Table 5 shows the relative importance of the two variables (smart governance and digital transformation) from the responses of a sample in selected Iraqi universities, as follows:

**Table 5.** Relative importance of the dimensions of the research variables.

Sequence	Variables	Arithmetic mean	Standard deviation	Response level%	Arrangement
1	Smart Governance	3.676	0.861	73.526	2
2	Digital Transformation	3.908	0.903	78.153	1

From Table 5 above, Digital transformation achieved a relatively higher edge (78.153%) over smart governance (73.526%), basically demonstrating the more important variable among the participants.

**Normal Distribution Test**: Table 6: Values of the (Kolmogorov-Smirnov) Criterion for Testing Normal Distribution.

**Table 6.** Test for the absence of multicollinearity.

Dimensions	Tolerance	VIF
Transparency	0.265	3.779
Participation	0.379	2.636
Accountability	0.266	3.753
Collaboration	0.547	1.829
Innovation	0.556	1.799

It is observed from the above Table 6 that the P-value for the two variables, Smart Governance and Digital Transformation, was equal to (0.000), which is less than (0.05). This indicates the rejection of the null hypothesis and the acceptance of the alternative hypothesis, which states that the two variables are not normally distributed.

**Test for the Absence of Multicollinearity:**

**Table 7.** Goodness-of-fit indices for Confirmatory Factor Analysis (CFA).

Indicators	Value	Result
CMIN/DF	0.39	identical
GFI	0.97	identical
AGFI	0.96	identical
PGFI	0.74	identical
NFI	0.96	identical
RFI	0.96	identical
RMR	0.06	identical

It is observed from Table 7 above that the Tolerance values were greater than (0.1). In addition, the Variance Inflation Factor (VIF) values were less than (10), indicating the absence of multicollinearity when examining the effect relationship between the dimensions of the Smart Governance variable and the Digital Transformation variable, according to Kim, J. H. (2019).

**Confirmatory Factor Analysis (CFA)**

Figure 1 below illustrates the Factor Loading values (correlations) between the observed variables (questions) and their corresponding latent variables related to the research variables.

**Figure 1.**
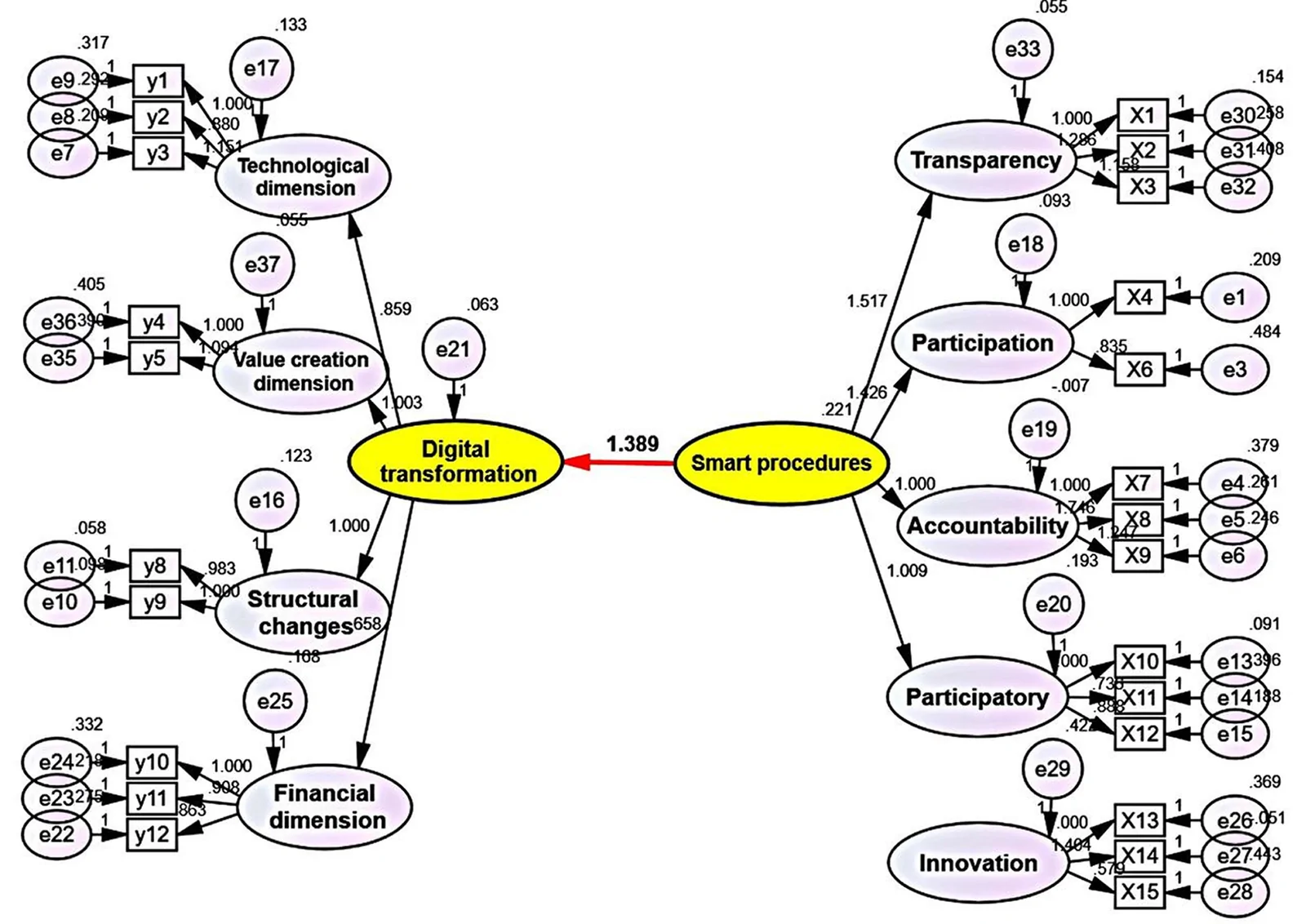
The impact relationship of the smart governance variable on the digital transformation variable.

**Goodness-of-Fit Indices:**

**Table 8.** Goodness-of-fit indices for Confirmatory Factor Analysis (CFA).

Indicators	Value	Result
CMIN/DF	0.30	identical
GFI	0.98	identical
AGFI	0.98	identical
PGFI	0.71	identical
NFI	0.98	identical
RFI	0.97	identical
RMR	0.051	identical

From Table 8 above it is evident that there is some weakness in the values of the Standardized Regression Weights (SRW), also referred to as Factor Loadings. Therefore, the items (y7, y6, X5) were deleted, with their corresponding values being (0.534, 0.588, 0.580).

**Goodness-of-Fit Indices After Deletion:**

Table 5 above shows that Digital Transformation ranked first in terms of relative importance, with a high response level of 78.153%, while Smart Governance ranked second with a response level of 73.526%. The results also indicate consistency in respondents’ opinions, as reflected by the low standard deviation values.

3. **Correlation and influence analysis of research variables**

**Correlation analysis**

The correlation coefficient is a measure that determines the strength and type of relationship between two variables. The correlation coefficient indicates the type of relationship between the variables, whether positive or negative. The correlation coefficient value also represents the strength of the relationship between them. The closer the correlation coefficient value is to one, whether positive or negative, the stronger the relationship between the two variables. Furthermore, the correlation coefficient may be significant or insignificant, which is determined by the P value. If the P value is less than 0.05, this indicates that the correlation coefficient is significant, and vice versa. Table 9 below shows the results of the correlation test between the research variables:

**Table 9.** Correlation between the smart governance variable and the digital transformation variable.

Independent variable	Relationship direction	Dependent variable	Link value	Confidence Interval 95%		P-value
				Lower	Upper	
Smart Governance	↔	Digital transformation	0.93	0.851	0.987	0.003

The correlation between the smart governance and the digital transformation is a direct relationship, as evidenced by the strong positive correlation coefficient value, which reached (0.93). The confidence interval (0.851 to 0.987), which translate to 95% and a Pvalue of 0.003 (<0.05) consolidate the statistical significance of this relationship. The correlation between the smart governance variable and the digital transformation variable can be observed in Figure 2:

**Figure 2.**
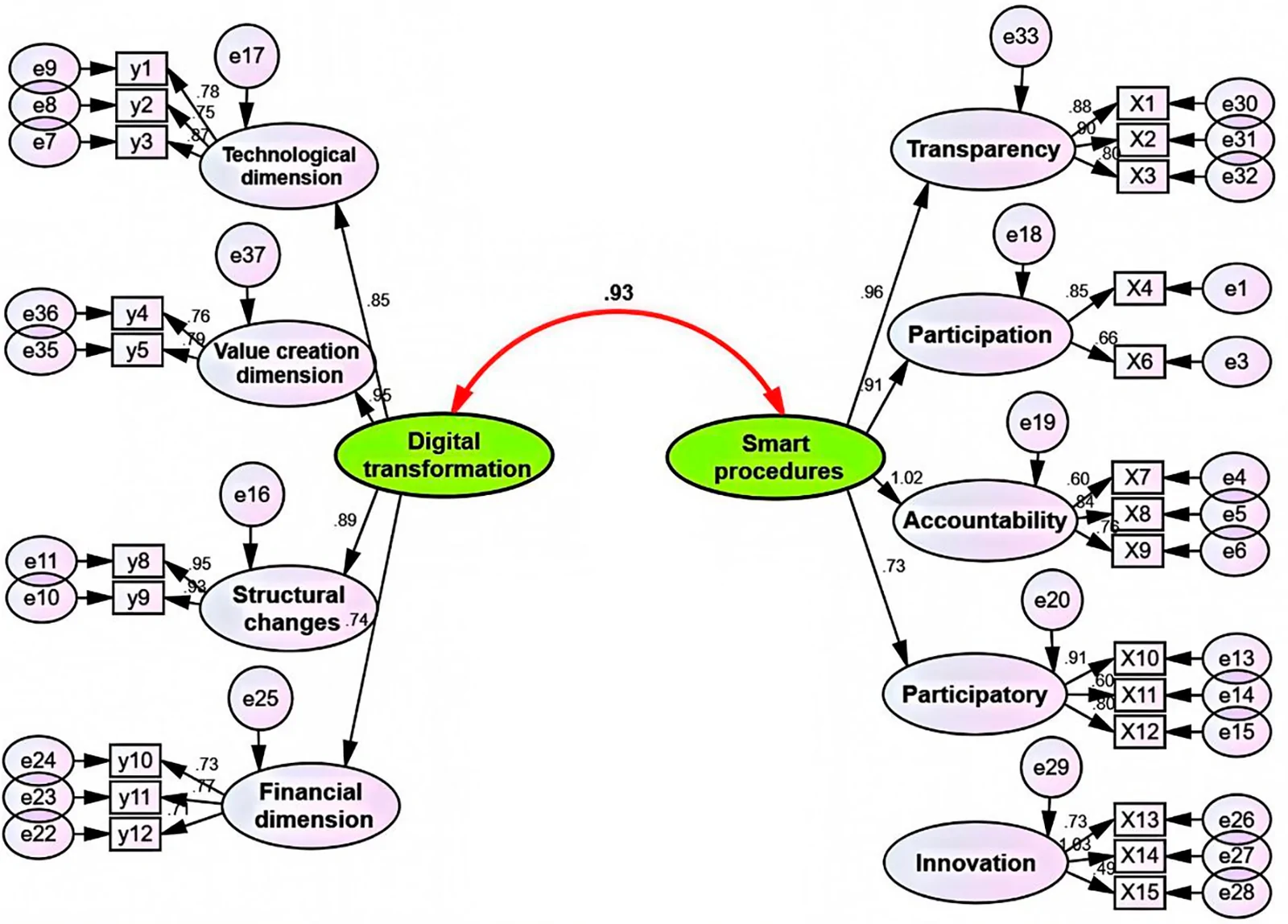
The correlation between the smart governance variable and the digital transformation variable.

**Impact analysis between research variables**. Table 10 below shows the results of the impact test between research variables.

**Table 10.** Regression Analysis of the Impact Between Variables.

Independent variable	Direction of influence	Dependent variable	Estimate(β)	Se.(β)	Confidence Interval 95%		P-value
					Lower	Upper	
Smart Governance	→	Digital transformation	1.389	0.529	2.789	0.741	0.002

The impact of the smart governance variable on the digital transformation variable is demonstrated by the estimated parameter attributed to the smart governance variable, which amounted to (1.389), indicating a direct relationship between the smart governance variable and the digital transformation variable, given the positive sign of this estimated parameter. The standard error value (S.E.) amounted to (0.529). NOTE: CONSIDER REWRITING THE YELLOW SECTION: The analysis signifies that smart governance, with an estimated parameter of (1.389), bears an influential significance on digital transformation. Furthermore, the impact of the smart governance variable on the digital transformation variable was statistically significant, as the p-value (0.002) appeared less than (0.05) for this relationship. The confidence interval (95%) appeared with similar signs, represented by the minimum and maximum limits (2.789 and 0.741), respectively. Therefore, based on the above results, a decision can be reached to accept the alternative hypothesis stating that the smart governance variable has an impact on the digital transformation variable. The impact of the smart governance variable on the digital transformation variable can be observed in Figure 3:

**Figure 3.**
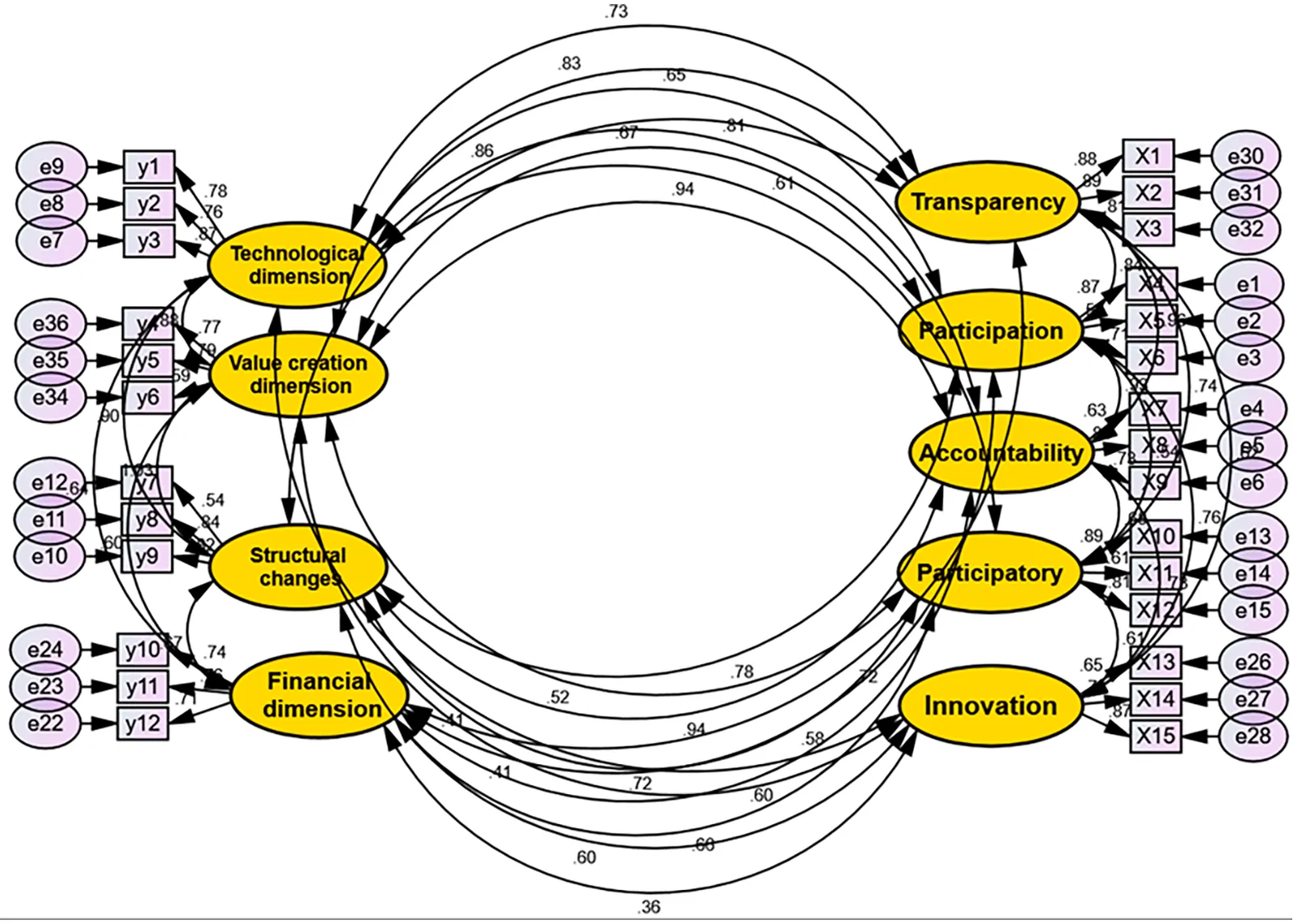
Confirmatory Factor Analysis of the Dimensions of the Study Variables.

## Discussion - Bridging the Research Gap - conclusions – recommendations

### Discussion

The findings indicate medium to high level smart governance in the surveyed universities with a clear tendency to follow the principles of smart governance which are transparency, participation, accountability and collaboration. The Creativity dimension, on the other hand, appeared at a lesser level, which shows that despite the importance of this dimension, there is still a need to improve the universities' capacity to include innovative thoughts in their activities and to find new mechanisms to support it. In the case of digital transformation, the results indicated that universities are making clear progress, especially in terms of technological infrastructure and the financial component, indicating that a serious effort is being made to invest in digital resources and technologies. At the same time, the size of value creation and structural transformation was at good level but needs additional support. The correlation analysis also showed a strong direct correlation between smart governance and digital transformation, which means that strengthening the governance principles directly contributes to accelerating and facilitating the digital transformation process. The findings of an impact analysis confirmed that smart governance is one of the essential elements that enable the implementation of digital transformation requirements, which is in line with the previous literature and research, which have shown that digital projects are more successful if they have effective and sound governance systems.

### Bridging the research gap

Unlike previous studies, this research aims to combine the concept of smart governance with the concept of digital transformation in the context of Iraqi universities, which has not been fully explored in past studies. The past studies focused mainly on the smart governance and digital transformation separately or in non-educational settings and in developed countries, but this research examined both variables in HEIs in Iraq.

The results enhance the existing knowledge by proving the role of smart governance dimensions such as transparency, participation, accountability, collaboration and Creativity in meeting the needs of digital transformation in universities. The research also includes empirical evidence from the perspective of university leaders, which can be used for practical action and provide useful insights into the ways in which governance practices can improve the readiness for digital transformation in developing educational environments.

## Conclusions

### Practical implications

The empirical evidence from this research offers several important policy recommendations for strengthening smart governance mechanisms and accelerating digital transformation in Iraqi higher education institutions. First, university leaders should establish integrated digital governance systems that unify administrative, academic, and financial processes on interoperable platforms, thereby enhancing transparency, traceability, and evidence-based decision-making. Second, it is essential to establish specialized digital transformation and smart governance units within universities, with clear mandates for strategic planning, implementation oversight, and performance evaluation.

In addition, the education sector needs to create and execute structured capacity building initiatives focused on building the advanced digital competencies of academic and administrative leaders, especially in data analytics, AI application and digital decision support systems. Alongside this, implementing e-accountability systems and performance dashboards in real-time is essential to increase transparency of institutions, increase responsiveness and align with strategic goals.

### Research limitations

Despite the contributions of this research, some methodological limitations must be acknowledged. First, the research is limited to a selected sample of academic leaders at specific Iraqi universities, which may limit the validity of the findings and their generalizability to other institutional or national contexts. Second, reliance on self-reported survey data may lead to methodological biases and common responses, potentially affecting the objectivity of the measured structures.

Furthermore, the cross-sectional design limits the ability to track the temporal dynamics of the development of smart governance practices and digital transformation. Therefore, future research is advised to employ longitudinal or mixed methodologies to enhance causal inference and deepen contextual understanding.

### Future research directions

Future studies should be encouraged to broaden the scope of analysis to include wider institutional samples, including public and private higher education institutions across different geographic regions. Comparative studies of governance contexts in developing and developed countries can help clarify the structural and contextual determinants of successful digital transformation.

Furthermore, incorporating qualitative methodologies, such as in-depth interviews and case research analysis, can provide deeper explanatory insights into governance processes and implementation challenges. Future research can also consider integrating additional explanatory variables, such as cybersecurity maturity, digital infrastructure readiness, and organizational culture, to develop a more comprehensive explanatory model of digital transformation effectiveness.

### Novelty and contribution

This research contributes to the literature by proposing and empirically validating a comprehensive conceptual framework that links the dimensions of smart governance transparency, participation, accountability, collaboration, and Creativity with the requirements of digital transformation in higher education institutions. The main novelty of this research lies in its contextual focus on Iraqi universities, an environment that remains underrepresented in empirical studies of governance and digital transformation.

Furthermore, the research contributes to theory by expanding the understanding of the interrelationship between governance and digital transformation within public sector institutions, and to practice by providing an evidence-based framework that can guide the formulation of strategic policies and institutional reform initiatives in higher education systems in developing countries.

## Recommendations

Universities hold the key to shaping innovation through prudent administration. By inspiring novel ideas and embracing emerging technologies, together with engaging stakeholders in crucial discussions, digital change can be steered fruitfully. Continued funding of technological infrastructure requires transparency to optimize limited means. Foremost, an overarching vision is needed—one connecting leadership fundamentals with transformation necessities, balancing progress responsibly. Collaborations within and beyond university walls likewise let experience be shared and the forefront of practice observed globally.

## Ethical considerations

This study involved human participants and was conducted in accordance with accepted ethical research standards and the principles outlined in the Declaration of Helsinki. Ethical approval was obtained from the Scientific Research Ethics Committee, University of Fallujah, Iraq (Approval No. HOF.HUM.2025.001). Written informed consent was obtained from all participants prior to their participation. All participants were informed about the purpose of the study, the voluntary nature of their participation, their right to withdraw at any time without consequences, and the confidentiality of their data.

## Informed consent

Participation in this study was voluntary. Before completing the questionnaire, participants were provided with a brief explanation of the study objectives and were informed that their responses would remain anonymous and confidential. By completing and submitting the questionnaire, participants indicated their informed consent to participate in the study.

## Data Availability

Repository name: Zenodo: Smart governance and its role in achieving digital transformation requirements: A survey study in selected Iraqi universities. https://doi.org/10.5281/zenodo.19019843 [ Ghareeb, et al. 2026]. The project contains the following underlying data:
• Data.xlsx (Raw data used for statistical analysis). • SPSS.sav (Dataset used for statistical analysis in SPSS). Repository name: Zenodo: Smart governance and its role in achieving digital transformation requirements: A survey study in selected Iraqi universities. https://doi.org/10.5281/zenodo.19019843 [ Ghareeb, et al. 2026]. This project contains the following extended data:
• Questionnaire.pdf (Survey instrument used for data collection). Data are available under the terms of the Creative Commons Zero “No rights reserved” data waiver (CC0 1.0 Public domain dedication). This study is an observational survey-based research and follows the STROBE reporting guidelines. No CONSORT or ARRIVE checklists are required, as the study does not involve clinical trials or animal experiments.
