# Supplementary material for: Smart governance and its role in achieving digital transformation requirements: A survey study in selected Iraqi universities
Source: F1000Res. 2026 Oct 5;15:538. Originally published 2026 Apr 16. [Version 2] doi: 10.12688/f1000research.174187.2 (PMC13202199; doi:10.12688/f1000research.174187.2)
Supplement: Peer review history

# Reviewer report: Reviewer response for version 2

**DOI:** 10.5256/f1000research.202826.r527472

**Published:** 2026-10-09

**Authors:** Eduardo Junio Andaya

**Affiliations:** 1 College of Public Administration and Governance, Pamantasan ng Lungsod ng Valenzuela (University of Valenzuela City), Valenzuela, Philippines

As of now, the author(s) have addressed all the necessary revisions and suggestions I previously made, leaving the paper clean and well-structured. In my opinion, it is now ready for indexing.

Is the work clearly and accurately presented and does it cite the current literature?

Partly

If applicable, is the statistical analysis and its interpretation appropriate?

Yes

Are all the source data underlying the results available to ensure full reproducibility?

Yes

Is the study design appropriate and is the work technically sound?

Partly

Are the conclusions drawn adequately supported by the results?

Partly

Are sufficient details of methods and analysis provided to allow replication by others?

Yes

Reviewer Expertise:

Public Administration, Social Sciences, e-Governance, Public Policy, Environmental Management, Sustainable Development

I confirm that I have read this submission and believe that I have an appropriate level of expertise to confirm that it is of an acceptable scientific standard.

# Reviewer report: Reviewer response for version 2

**DOI:** 10.5256/f1000research.202826.r527470

**Published:** 2026-10-09

**Authors:** Andi Cudai Nur

**Affiliations:** 1 Tourism, Indonesia Open University, Makassar, South Sulawesi, Indonesia; 2 Administration Science, University of Negeri Makassar, Makassar, South sulawesi, Indonesia

The methodology section requires revision to specify that the research is quantitative-descriptive in nature; furthermore, the methodology should align cohesively with the results and conclusions regarding smart governance, digital transformation, and sustainable development.

Is the work clearly and accurately presented and does it cite the current literature?

Yes

If applicable, is the statistical analysis and its interpretation appropriate?

Yes

Are all the source data underlying the results available to ensure full reproducibility?

Yes

Is the study design appropriate and is the work technically sound?

Yes

Are the conclusions drawn adequately supported by the results?

Yes

Are sufficient details of methods and analysis provided to allow replication by others?

Yes

Reviewer Expertise:

Public Administration, Tourism,  Environmental Policy, and SMEs

I confirm that I have read this submission and believe that I have an appropriate level of expertise to confirm that it is of an acceptable scientific standard, however I have significant reservations, as outlined above.

# Reviewer report: Reviewer response for version 1

**DOI:** 10.5256/f1000research.192069.r482653

**Published:** 2026-05-26

**Authors:** Isagani M Tano

**Affiliations:** 1 Quezon City University, Quezon City, Philippines

The article investigates the role of smart governance in achieving digital transformation requirements in selected Iraqi universities. It focuses on smart governance dimensions such as transparency, participation, accountability, participatory governance, and innovation, and relates them to digital transformation dimensions such as technology, value creation, structural change, and finance. Using a descriptive-analytical survey design, the study collected questionnaire data from 83 university leaders from Anbar University, Fallujah University, and Al-Maaref University. The results indicate that digital transformation received a higher relative importance score than smart governance, and that smart governance was strongly and positively correlated with digital transformation . The topic is timely and relevant, especially because universities increasingly rely on digital systems for academic and administrative work.

 The article has several strengths. It addresses an important issue in higher education administration, focuses on a specific national context, includes university leaders as respondents, and provides open access to the underlying data and questionnaire. The theoretical framework discusses relevant dimensions of smart governance and digital transformation, and the results are presented through tables and figures that help readers understand the main findings. The inclusion of ethical approval and informed consent information is also a positive feature, as the manuscript states that approval was obtained from the Scientific Research Ethics Committee of the University of Fallujah with approval number HOF.HUM.2025.001 .

 However, several points must be addressed to make the article scientifically sound. First, the manuscript needs thorough language editing and formatting correction. The authors should remove internal notes, correct awkward phrasing, standardize terminology, and ensure that table labels and variable names are consistent. For example, the manuscript alternates between “innovation” and “creativity,” and “collaborative” and “participatory,” which may confuse readers. Second, the literature review should be strengthened by adding more recent and reputable studies, especially empirical studies on smart governance and digital transformation in higher education. The authors should also remove irrelevant or misplaced citations and ensure that the reference list follows a consistent style.

 The methodology also requires improvement. The authors should clearly explain how the questionnaire was developed, how many items measured each dimension, how each variable was scored, and how validity and reliability were assessed. Although the manuscript mentions expert validation and pilot testing, it does not provide enough detail on the results of these procedures. The authors should report reliability coefficients, preferably Cronbach’s alpha or composite reliability, for each construct. They should also clarify whether the questionnaire items were adapted from previous instruments or newly developed for this study.

 The statistical section needs careful revision. The authors should check all reported values, especially the regression confidence interval, because the lower and upper bounds appear to be incorrectly ordered or inaccurately calculated. They should also explain the assumptions behind the correlation and regression analyses, such as linearity, normality, independence, and absence of multicollinearity. If the figures represent a path model or structural equation model, then the authors must report model fit indices and explain the estimation procedure. Without these details, the statistical analysis is only partly verifiable from the manuscript text.

 The conclusion should be revised to avoid overclaiming. The current results support a strong relationship between smart governance and digital transformation, but they do not prove that smart governance guarantees successful digital transformation or sustainable development. The authors should limit their claims to what the study actually measured: perceptions of university leaders regarding governance practices and digital transformation requirements. They should also add a limitations section discussing the cross-sectional design, self-report data, limited institutional scope, and possible response bias. Finally, the recommendations should be more specific, such as strengthening innovation mechanisms, improving participatory decision-making platforms, developing digital governance policies, and conducting future studies with larger samples and objective institutional performance indicators.

 Overall, the paper has indexing potential, but it requires revision before it can be considered fully scientifically sound. The most important required revisions are statistical verification, clearer methodological reporting, stronger literature integration, correction of citation and formatting issues, and more cautious interpretation of the findings.

Is the work clearly and accurately presented and does it cite the current literature?

Partly

If applicable, is the statistical analysis and its interpretation appropriate?

Partly

Are all the source data underlying the results available to ensure full reproducibility?

Yes

Is the study design appropriate and is the work technically sound?

Partly

Are the conclusions drawn adequately supported by the results?

Partly

Are sufficient details of methods and analysis provided to allow replication by others?

Partly

Reviewer Expertise:

Information Technology, Computer Science, Education Leadership, Management, Public Administration

I confirm that I have read this submission and believe that I have an appropriate level of expertise to confirm that it is of an acceptable scientific standard, however I have significant reservations, as outlined above.

# Response

**Published:** 2026-05-29

**Authors:** Ahmed Ghareeb

**Affiliations:** University of Fallujah, Al-Fallujah, Al Anbar Governorate, Iraq

Thank you for your valuable comments and constructive suggestions. Your observations were very helpful in improving the quality and clarity of the manuscript. I have carefully considered all your comments and addressed them in the revised version of the paper.

**Comments of Reviewer 2**

1. **the study method and design:**

The “Study Methodology and Design” section was revised to clearly distinguish between the study method and the study design. The quantitative method is now presented first, followed by the descriptive-analytical design in accordance with established methodological standards.

1. **clarification of data types:**

The manuscript was revised to provide a clearer explanation of the data sources used in the study. Specifically, primary data collected through the questionnaire and secondary data obtained from previous studies, academic journals, books, and scholarly references related to the study variables were clearly identified.

1. **inclusion of the study gap in the abstract:**

A clear statement of the study gap was added to the abstract, highlighting the limited number of studies examining the relationship between smart governance and digital transformation requirements within Iraqi universities from the perspective of university leaders. The contribution of the current study in addressing this gap was also clarified.

1. **clarification of the study objectives:**

The study objectives were reorganized and presented more clearly and systematically, including objectives related to measuring the level of smart governance practices, assessing digital transformation requirements, and examining the relationship and impact between the two variables.

1. **ethical approval:**

A separate section entitled “Ethical Considerations” was added to the manuscript. This section clarifies that official ethical approval was obtained from the Scientific Study Ethics Committee at the University of Fallujah, including the approval number. It also explains that informed consent was obtained from participants, while confidentiality and voluntary participation were ensured.

1. **clarification of how the study addresses the study gap:**

A separate subsection entitled “Bridging the Study Gap” was added to explain how the findings contribute to addressing the identified gap in the literature and how the study extends current knowledge, particularly within the context of Iraqi universities.

1. **the role of theories in supporting the study:**

An explanatory section was added to clarify the role of Stakeholder Theory and Resource Theory in supporting the conceptual framework of the study and explaining the relationship between smart governance and digital transformation.

1. **additional cross-referencing of previous studies:**

The literature review section was strengthened by adding further analytical cross-referencing among recent related studies. Similarities, differences, and complementary findings between studies on smart governance and digital transformation were clarified.

1. **conclusions and recommendations:**

The conclusion section was substantially improved by including:

- Practical implications and policy recommendations for enhancing smart governance and accelerating digital transformation in Iraqi universities.
- Study limitations related to sample size, data collection method, and generalizability.
- Future study directions, including broader samples, longitudinal and qualitative approaches, and the inclusion of additional explanatory variables.

1. **the novelty and contribution of the study:**

A separate subsection was added to clarify the novelty and academic contribution of the study. The manuscript now clearly explains that the study develops and empirically validates a conceptual framework linking smart governance dimensions with digital transformation requirements within the underexplored context of Iraqi universities.

1. **references and citation style:**

The entire reference list was carefully reviewed, updated, and reformatted according to APA 7th edition requirements. Additional recent and high-quality scholarly references were also incorporated to strengthen the theoretical and analytical foundations of the study.

# Reviewer report: Reviewer response for version 1

**DOI:** 10.5256/f1000research.192069.r482658

**Published:** 2026-05-22

**Authors:** Eduardo Junio Andaya

**Affiliations:** 1 College of Public Administration and Governance, Pamantasan ng Lungsod ng Valenzuela (University of Valenzuela City), Valenzuela, Philippines

**Title**

 Comments:

 1. The title clearly states the study's methodology and target group.

 2. The title avoids using technical jargon that might be difficult for a general academic audience to understand.

**Abstract:**

 Comments:

 1. The abstract is clear and accurately summarizes the study's findings. It is well within the 300-word limit.

 2. The abstract clearly highlights the topic's significance and identifies a major gap: despite Antique Province’s high vulnerability to recurring natural disasters, a significant research gap exists regarding the specific disaster recovery and resiliency strategies of its resort sector. 

 3. The study's objective is clearly stated

 4.  The abstract offers a compelling conclusion with specific recommendations

 Recommendation:

 1. Please make sure to present your method first, followed by the design. The method is different from the design, and both should be addressed in the initial statement of the Research Design section. 

 2. Please include the gap(s) on the abstract section

**Introduction:**

**Comment(s)**

 1. Start with a broad theme or topic for the study.

 2. In the introduction, the researcher(s) address the crucial question, "Why is this article important?" 

 3. The researcher(s) indicated the most important gap and theoretical framework.

 4. The ideas are well-structured and transition smoothly

 Recommendation(s): 

 1. Please include the concrete objectives of the study.

**Methodology:** 

 Comment(s):

 1. Participants and Sampling Technique: This section provides a detailed description of the study's participants.

 2. Clearly stated the research instrument, Data Gathering, and data analysis procedures

 Recommendation(s):

 1. I understand the researchers' perspective on the type of design they used, but please make sure to present your method first, followed by the design. The method is different from the design, and both should be addressed in the initial statement of the Research Design section.

 2. The researcher(s) did not clarify the types of data required, whether they were primary or secondary.

 3. Please include the ethical clearance from the Institutional or ethical review board with the code or serial number.

**Analysis of Results**

 Comment(S)

 1. The manuscript clearly presents the data through well-referenced tables with good interpretation, following the protocol on how to put the cross-reference to support the results. The transition from descriptive statistics to inferential results is logical, providing the reader with a comprehensive understanding. There is no significant repetition of data; instead of merely restating the numbers, the text effectively interprets the information displayed in the tables.

 2. The manuscript effectively combines the results and discussion into one cohesive section. Each finding is promptly followed by an explanation of its significance and is contextualized with references to relevant studies.

 3. The section is titled "RESULTS and DISCUSSION," which integrates both aspects. This format aligns well with the journal's requirement for a combined approach.

 4. Good to mention that the theories presented in the Introduction section help the study to establish knowledge. It connects the study to existing literature and scholarly traditions. It shows that this study work is not just a random collection of observations, but is grounded in proven concepts.

 Recommendation(s):

 1. The researcher(s) discussed how to address the gap; however, please separate the discussion on how findings address a gap in the literature, extend current knowledge, or provide new insights into a specific phenomenon or problem.

 2. Please explain how your theories help your study.

 3. Add more cross-references from your review of related literature.

**Conclusions**

 Comment(s)

 1. The researcher(s) clearly explained the key conclusions in the summary.

 Recommendation/s:

 1. But, I’m a little satisfied with the future works. Please follow this step to improve the conclusions:

 •First, it would be beneficial for the authors to outline some practical implications of their findings, specifically what actions can be taken to enhance community preparedness in the areas addressed in this manuscript. They should provide specific recommendations rather than relying on general statements.

 •In doing so, the authors must be specific about the policy actions that could be undertaken. 

 •Second, the authors could identify the limitations of their research, which may including sample size issues or response bias.

 •This would be useful for a reader who wants to know whether the results are generalizable to a different community. 

 •Finally, the authors can offer insights into potential future research activities, focusing on how to advance or extend the findings discussed in this manuscript.

 2. What is the novelty of this study, and how does it contribute to the advancement of knowledge in this field?

**References**

 Please ensure that you adhere to the APA 7th Edition guidelines when formatting the reference section.

Is the work clearly and accurately presented and does it cite the current literature?

Partly

If applicable, is the statistical analysis and its interpretation appropriate?

Yes

Are all the source data underlying the results available to ensure full reproducibility?

Yes

Is the study design appropriate and is the work technically sound?

Partly

Are the conclusions drawn adequately supported by the results?

Partly

Are sufficient details of methods and analysis provided to allow replication by others?

Yes

Reviewer Expertise:

Public Administration, Social Sciences, e-Governance, Public Policy, Environmental Management, Sustainable Development

I confirm that I have read this submission and believe that I have an appropriate level of expertise to confirm that it is of an acceptable scientific standard, however I have significant reservations, as outlined above.

# Response

**Published:** 2026-05-29

**Authors:** Ahmed Ghareeb

**Affiliations:** University of Fallujah, Al-Fallujah, Al Anbar Governorate, Iraq

Thank you for your valuable comments and constructive suggestions. Your observations were very helpful in improving the quality and clarity of the manuscript. I have carefully considered all your comments and addressed them in the revised version of the paper.

**Comments of Reviewer 3**

1. **Language, terminology, and formatting revisions:**

- The manuscript underwent comprehensive language editing and formatting correction.
- Terminology was standardized throughout the manuscript to avoid inconsistency. Specifically) Innovation” was adopted instead of “Creativity”.,Collaborative” was replaced with “Participatory” where appropriate).
- Internal notes and inconsistent labels were removed.

1. **Literature review enhancement:**

- The literature review was strengthened by incorporating recent and reputable empirical studies related to smart governance and digital transformation, particularly in higher education and smart systems contexts.
- Additional recent references from high-quality journals were integrated into the theoretical framework.
- The reference list was revised and fully standardized according to APA 7th edition formatting requirements.

1. **Methodological clarification:**

- Additional methodological details were included regarding questionnaire construction, dimensions measurement, scoring procedures, and validation processes.
- The manuscript now clarifies the expert validation and pilot testing procedures in greater detail.

1. **Statistical analysis revision:**

- All statistical values were carefully rechecked and verified.
- The upper and lower limits were confirmed, as they were found using the re-sampling method since the data did not follow a normal distribution, and it was found that the upper and lower limits were correct and there was no particular error in them.
- The assumptions underlying correlation and regression analyses were explicitly addressed.

1. **Assumption testing:**

**The following diagnostic tests were added and reported:**

- Normality test using Kolmogorov–Smirnov.
- Multicollinearity diagnostics using Tolerance and Variance Inflation Factor (VIF).

**The results confirmed:**

- Non-normal distribution of variables.
- Absence of multicollinearity problems among the dimensions.

1. **Confirmatory Factor Analysis (CFA)**

- A detailed Confirmatory Factor Analysis was conducted and added to the manuscript.
- Model fit indices including CMIN/DF, GFI, AGFI, PGFI, NFI, RFI, and RMR were reported and interpreted.
- Items with weak standardized regression weights (loading factors) were removed to improve model fit quality.

1. **Conclusions and interpretation:**

- The conclusion section was revised to avoid overgeneralization and causal overclaiming.
- Interpretations were limited to the actual scope of the study and respondents’ perceptions.
- Recommendations were refined to be more specific and practically applicable.

# Reviewer report: Reviewer response for version 1

**DOI:** 10.5256/f1000research.192069.r482657

**Published:** 2026-05-12

**Authors:** Andi Cudai Nur

**Affiliations:** 1 Tourism, Indonesia Open University, Makassar, South Sulawesi, Indonesia; 2 Administration Science, University of Negeri Makassar, Makassar, South sulawesi, Indonesia

This article is very interesting, presented clearly and accurately, and cites appropriate literature. However, it would be better to also cite articles from recent, reputable journals Q1 and Q2.

 The research design is appropriate and technically sound. However, Turnitin is recommended to avoid plagiarism.

 The details of the methods and analysis provided are sufficient and allow for replication by others, as many dimensions are presented and further testing can be performed.

 The statistical analysis and interpretation are appropriate.

 Advice from a qualified statistician is welcome.

 All source data underlying the results are available for reproducibility.

 The conclusions drawn are adequately supported by data analysis. If additional findings are found in the field, please include them.

Is the work clearly and accurately presented and does it cite the current literature?

Yes

If applicable, is the statistical analysis and its interpretation appropriate?

Yes

Are all the source data underlying the results available to ensure full reproducibility?

Yes

Is the study design appropriate and is the work technically sound?

Yes

Are the conclusions drawn adequately supported by the results?

Yes

Are sufficient details of methods and analysis provided to allow replication by others?

Yes

Reviewer Expertise:

Public Administration, Tourism,  Environmental Policy, and SMEs

I confirm that I have read this submission and believe that I have an appropriate level of expertise to confirm that it is of an acceptable scientific standard.

# Response

**Published:** 2026-05-29

**Authors:** Ahmed Ghareeb

**Affiliations:** University of Fallujah, Al-Fallujah, Al Anbar Governorate, Iraq

Thank you for your valuable comments and constructive suggestions. Your observations were very helpful in improving the quality and clarity of the manuscript. I have carefully considered all your comments and addressed them in the revised version of the paper.

 The comments have been taken into consideration, and additional references from Scopus-indexed Q1 journals have been added.
